# The Effects of Pain and Analgesic Medications on Blood Pressure

**DOI:** 10.1007/s11906-022-01205-5

**Published:** 2022-06-15

**Authors:** Giulia Rivasi, Silvia Menale, Giada Turrin, Antonio Coscarelli, Antonella Giordano, Andrea Ungar

**Affiliations:** Hypertension Clinic, Syncope Unit, Division of Geriatric and Intensive Care Medicine, University of Florence and Azienda Ospedaliero Universitaria Careggi, Largo Brambilla 3, 50134 Florence, Italy

**Keywords:** Opioids, Paracetamol, NSAIDs, Hypertension, Blood pressure control

## Abstract

**Purpose of Review:**

To review the blood pressure (BP) effects of pain and analgesic medications and to help interpret BP changes in people suffering from acute or chronic pain.

**Recent Findings:**

Acute pain evokes a stress response which prompts a transient BP increase. Chronic pain is associated with impaired regulation of cardiovascular and analgesia systems, which may predispose to persistent BP elevation. Also analgesics may have BP effects, which vary according to the drug class considered. Data on paracetamol are controversial, while multiple studies indicate that non-steroidal anti-inflammatory drugs may increase BP, with celecoxib showing a lesser impact. Hypotension has been reported with opioid drugs. Among adjuvants, tricyclic antidepressants and serotonin-norepinephrine reuptake inhibitors could be pro-hypertensive due to potentiation of adrenergic transmission.

**Summary:**

Pain and analgesics may induce a clinically significant BP destabilization. The implications on hypertension incidence and BP control remain unclear and should be explored in future studies.

**Graphical abstract:**

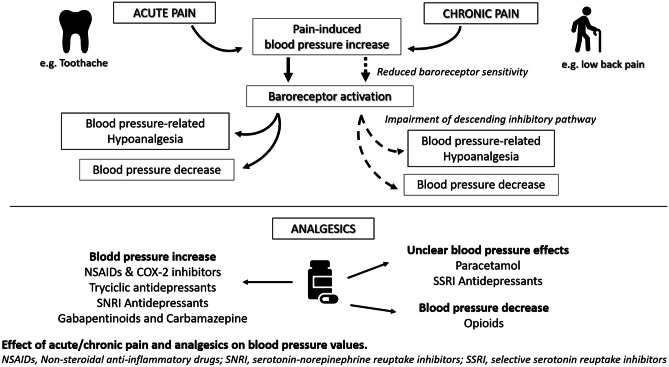

## Introduction

Pain is defined as “an unpleasant sensory and emotional experience, associated with actual or potential tissue damage” [1]. According to its time course, it is commonly classified into acute and chronic pain. Acute pain is a physiologic response to noxious stimuli; it is sudden in onset and time-limited, lasting for less than 3 months. Chronic pain persists past normal healing time, lasting or recurring for more than 3 to 6 months [1].

Pain is a very frequent health complaint and represents one of the most common reasons for adults seeking medical care, particularly among older individuals. Acute pain is highly prevalent in the primary care, with low back pain and headache representing the most frequent causes [2]. It is also common in the hospital setting, where up to 80% of patients complain of pain, which is reported to be severe in 9–36% of cases [3]. Chronic pain affects 25–35% of people worldwide showing increasing prevalence with advancing age. It represents one of the major causes of depression, poor quality of life, mobility restriction, and disability, thus significantly affecting individuals’ psychosocial well-being [4, 5].

Similarly to pain, hypertension is highly prevalent in the general and geriatric population [6], so that hypertension and pain frequently coexist in clinical practice [7•]. Both pain and analgesic medications are known to affect blood pressure (BP) values, with pressor effects varying according to pain duration and the drug class considered [8, 9••, 10••]. Consequently, pain and analgesics may potentially influence the development of arterial hypertension and interfere with BP control in hypertensive patients. The knowledge of the BP effects of pain and analgesics could thus be useful in the context of hypertension management.

This narrative review provides an overview of available evidence concerning the effects of pain and analgesic medications on BP (Table 1), which may be helpful to interpret BP changes in patients suffering from acute or chronic pain.

**Table 1:** Effects of most commonly prescribed analgesics on blood pressure

**Analgesics**	**Effects on BP values**	**Mechanism potentially responsible for blood pressure changes**
*Paracetamol*	Unclear	Inhibition of prostaglandins synthesis [10••, 28] Vasodilation [29] Sodium content of effervescent formulations [30]
*NSAIDs and COXIB*	BP increase	Inhibition of prostaglandins synthesis [45] Increased production of endothelin-1, increased levels of aldosterone [46, 47] Calcium-mediated reduced vascular tone (celecoxib) [49]
*Opioids*	BP decrease	Histamine release and histamine-mediated vasodilation [57–59] Reduced sympathetic tone [62]
*Antidepressants* TCA SNRI SSRI	BP increase BP increase Unclear	Potentiation of adrenergic transmission, anticholinergic effects [72, 73•] Potentiation of adrenergic transmission [72] Vasodilation, reduced sympathetic activity, cytochrome inhibition [74, 77, 86]
*Anticonvulsants* Gabapentinoids Carbamazepine	Possible BP increase	Activation of the nitric oxide pathway (gabapentinoids) [80] Antagonism of central noradrenergic transmission (carbamazepine) [82–84] Cytochrome induction and increased metabolism of antihypertensive medications (carbamazepine) [85]

*BP* blood pressure, *NSAIDs* non-steroidal anti-inflammatory drugs, *TCA* trycyclic antidepressants, *SNRI* serotonin-noradrenalin reuptake inhibitors, *SSRI* selective serotonin reuptake inhibitors

## Physiology of Pain and Analgesia

**Fig. 1:**
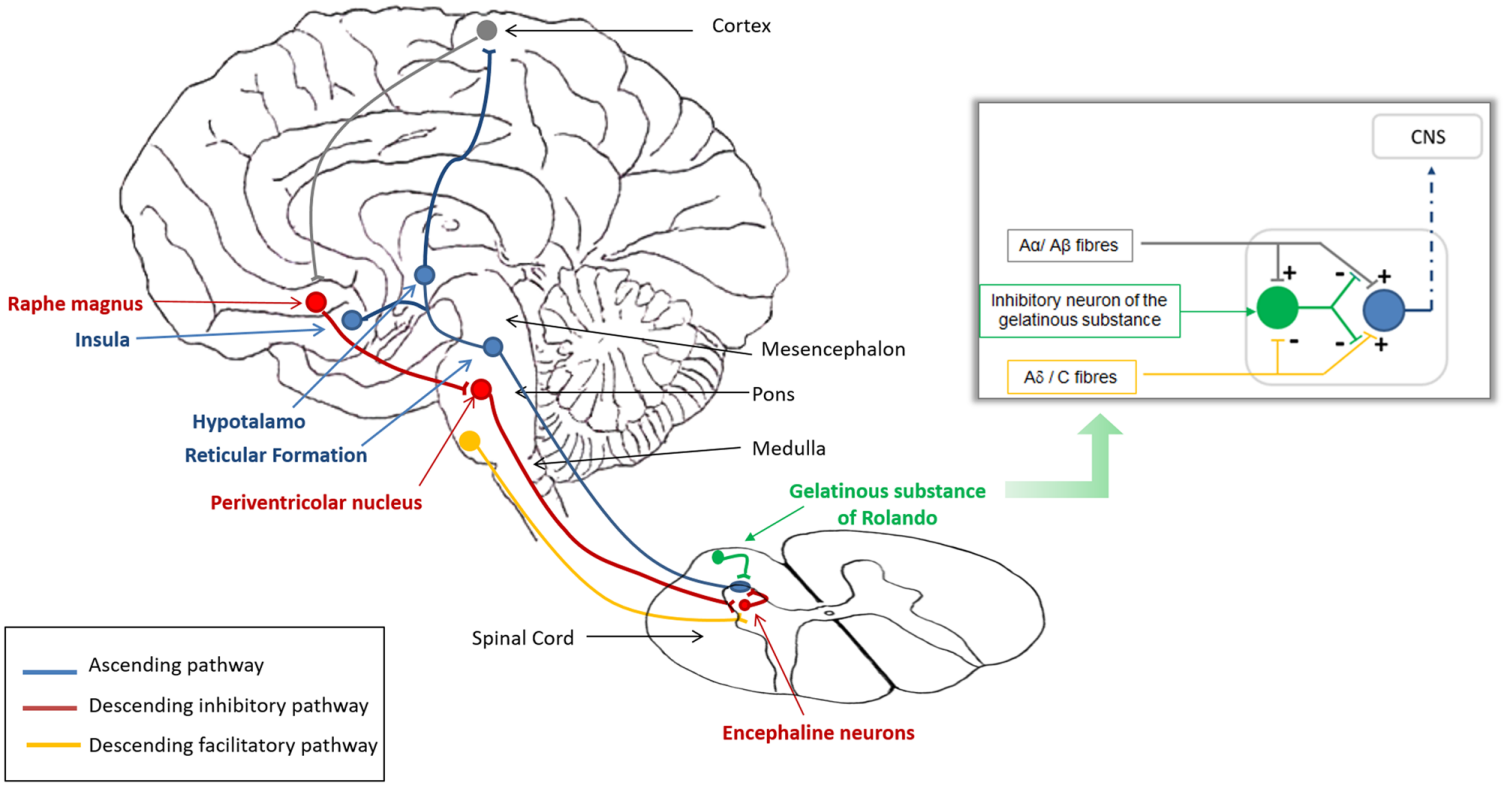
Physiology of pain and analgesia. CNS, central nervous system

Somatic nociceptive stimuli enter the spinal cord through the dorsal roots of the spinal nerves and are transmitted along myelinated Aδ and C fibers of the anterolateral system. These afferent fibers synapse in the dorsal horns of the spinal gray matter, and then cross to the opposite side and ascend through the anterior and lateral white columns of the cord up to the thalamus. Nociceptive stimuli are then transmitted to the cortex and subcortical areas, which activate the descending pathways [11]. In the central nervous system, areas playing a major role in pain perception and codification include the reticular formation (integration of pain experiences), the limbic system (emotional responses to pain), the hypothalamus (vegetative and neuroendocrine responses to pain), and the thalamus (pain awareness and subsequent reactions) (Fig. 1) [8].

Pain perception is modulated at a central level through descending inhibitory pathways referred to as the “analgesia system,” consisting of a network of inhibitory neurons which suppress pain signals before they are relayed to the central nervous system. Its main centers are located in the periaqueductal and periventricular areas of the mesencephalon and in the medulla, which activate the inhibitory neurons of the dorsal horns of the spinal cord (Fig. 1) [12]. Several neurotransmitters are involved, particularly encephalin and serotonin, which mediate a presynaptic and postsynaptic inhibition of Aδ and C fibers in the dorsal horns, so that pain stimuli are blocked immediately after entering the spinal cord. Pain perception is also modulated at the spinal level by the “gelatinous substance of Rolando,” which inhibits the transmission of incoming nociceptive stimuli along Aδ and C fibers. This mechanism is known as “gate control” and can be activated also by tactile stimuli, thus explaining why simple maneuvers such as rubbing the skin may provide pain relief.

In contrast to the analgesia system, a facilitating descending pathway exists, which originates at a supraspinal level and facilitates the relay of pain stimuli to the brain (Fig. 1). It constitutes a defense mechanism, aimed at inducing the individual to escape potentially harmful situations. An abnormal, persistent activation of the facilitating pathway may occur in some pathological conditions, leading to chronic pain and hyperalgesia, as it is supposed to happen in chronic muscle pain, neuropathic pain, and migraine [13].

## Pain and Blood Pressure

**Fig. 2:**
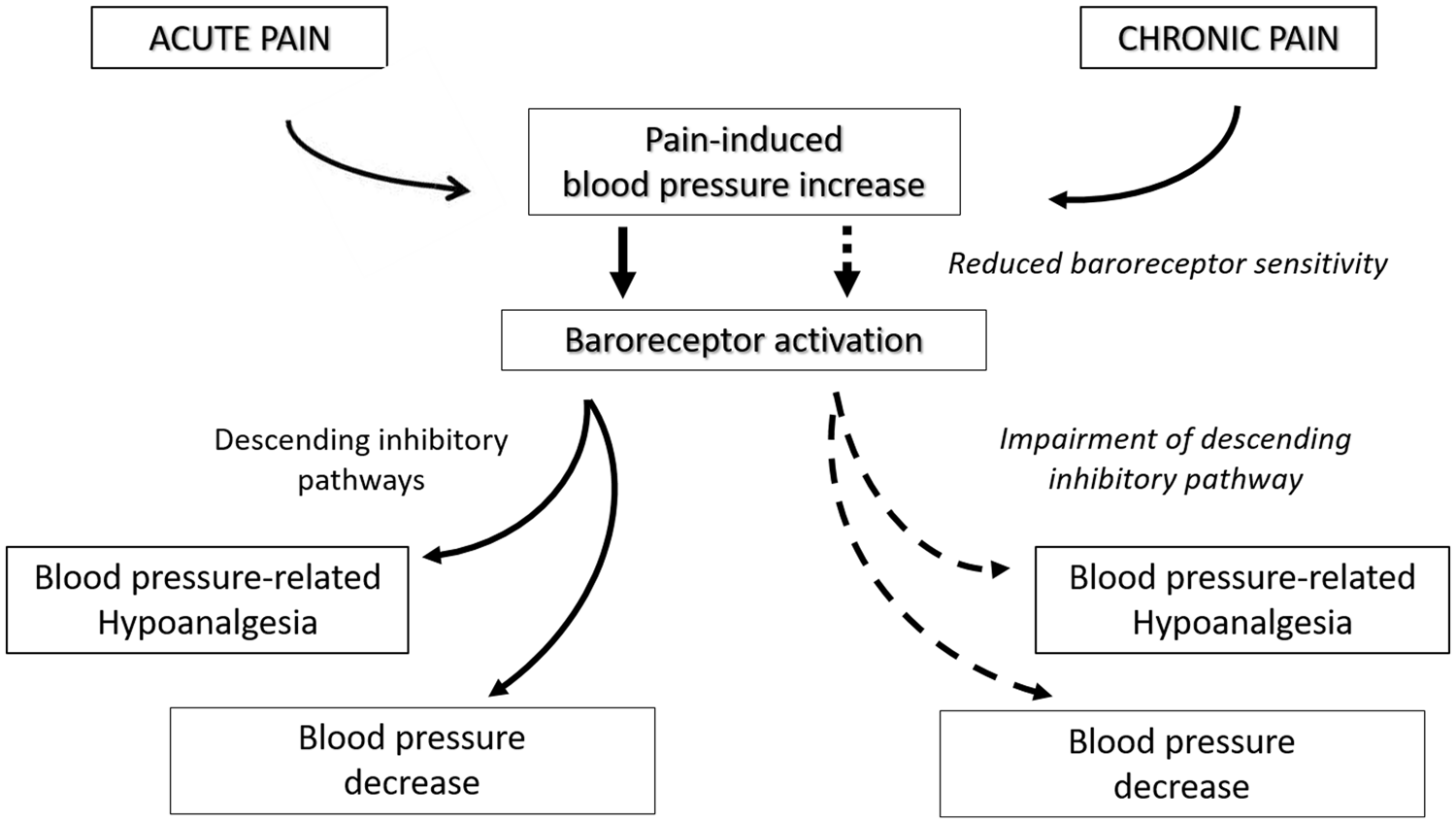
The effects of acute and chronic pain on blood pressure

Pain is associated with neuro-endocrine and autonomic responses that can raise BP. Indeed, pain evokes a stress response involving the activation of the hypothalamic–pituitary–adrenal axis and the sympathetic nervous system, which integrate and potentiate each other [14]. The subsequent release of cortisol and catecholamines results in a BP increase, which is detected by carotid and aortic baroreceptors involved in BP regulation. Experimental studies indicate that baroreceptors not only induce a compensatory BP reduction but also activate the analgesia system, with the final aim to suppress pain stimuli and restore the homeostasis (Fig. 2). Indeed, baroreflex activation was shown to reduce pain perception (the so-called “BP-related hypoalgesia”) and may also affect pain-related anxiety and avoidance behaviors [14, 15].

The baroreceptor response to pain-induced BP elevation tends to wane in chronic pain conditions, probably due to decreased vagal inhibitory activity or baroreceptor desensitization [16–18•]. Consequently, chronic pain may prompt a persistent elevation in BP values, resulting from the failure of homeostatic control mechanisms (Fig. 2). Additionally, chronic pain may determine a dysfunction or a progressive exhaustion of descending inhibitory pathways [12, 16, 19], while the facilitating pathway continues to promote transmission of nociceptive information and may also be over-activated, thus further increasing pain sensitivity [12].

Given the above, we might expect that chronic pain is associated with an increased risk of hypertension [18•]. Similarly, we may hypothesize that chronic pain interferes with BP lowering in hypertensive individuals, thus predisposing to poor BP control. In a study by Bruehl et al. patients complaining of chronic pain had significantly higher prevalence of hypertension as compared to controls (39% vs 21%, *p* = 0.001) and pain intensity was an independent predictor of hypertension [7•]. Yet, data regarding incidence of hypertension in chronic pain sufferers are lacking [16]. Likewise, the impact of chronic pain on BP control in hypertensive subjects has not been investigated to date. In view of the epidemiological relevance of chronic pain, it is desirable that future studies explore its role as a potential risk factor for both hypertension and poor BP control.

## Analgesic Medications and Blood Pressure

### Paracetamol

Paracetamol is included among over-the-counter medications and represents a first-line therapy for both acute and chronic pain. In hypertensive individuals, paracetamol is commonly considered to be a safer alternative to non-steroidal anti-inflammatory drugs (NSAIDs), as it is supposed to have limited impact on BP values. Yet, evidence concerning the effects of paracetamol on BP is weak and controversial.

Previous observational studies reported a higher risk of incident hypertension in patients using paracetamol compared with non-users, with a trend suggesting increasing risk with increasing frequency of use [20–22]. By contrast, no risk increase was reported in paracetamol users by Kurth et al. [23]. However, the observational design of these studies limits the interpretation of causal links.

Prospective, randomized controlled trials assessing the effects of paracetamol on BP are scarce and report conflicting results [10••]. Radack et al. did not observe any significant BP change in hypertensive patients receiving paracetamol 1 g three times a day [24]. A study by Pavlicevic´ et al. [25] compared the BP effects of ibuprofen (400–600 mg, 3/day) and piroxicam (10–20 mg/day) followed by paracetamol (1 g, 3/day) in hypertensive patients and controls receiving either lisinopril/hydrochlorothiazide or amlodipine. In the lisinopril/hydrochlorothiazide subgroup, both ibuprofen and piroxicam led to a significant BP increase, while a BP decrease was observed during the paracetamol phase, suggesting a hypotensive effect. However, these findings were not confirmed in the amlodipine subgroup. A randomized placebo-controlled study in patients with coronary artery disease observed a significant increase in ambulatory BP after 2 weeks’ paracetamol treatment (1 g, 3/day), with systolic and diastolic BP varying from 122 to 125 mmHg and from 73 to 75 mmHg, respectively (*p* < 0.02 for both) [26]. Similarly, a randomized, double-blind placebo controlled trial by Chalmers et al. reported a 4 mmHg increase in systolic BP in a small sample of hypertensive patients receiving paracetamol 1 g three times a day [27].

Although widely prescribed, the pharmacodynamics of paracetamol remains largely unknown. It is assumed that it acts through the inhibition of the cyclo-oxygenase (COX) pathway, thus reducing the synthesis of prostaglandins (PGs) which mediate inflammation and pain. It is plausible that the potential effects of paracetamol on BP are related to the inhibition of PGs production, with particular reference to PGE2 and prostacyclin (PGI2). Indeed, both these molecules are potent vasodilators and their lower levels might allow for the predominance of vasoconstrictor substances such as endothelin. Additional effects of PGI2 and PGE2 include stimulation of natriuresis and attenuation of norepinephrine release [28, 29], which may contribute to explain the hypertensive effect of paracetamol reported by some studies.

In addition to the above, the salt content of paracetamol may potentially be responsible for BP changes, as effervescent formulations contain significant amounts of sodium bicarbonate [10••]. Consistently, the shift from effervescent formulations to sodium-free tablets was found to induce a clinically significant BP decrease in older hypertensive patients (− 13 mmHg, *p* < 0.0001, and − 2.5 mmHg, *p* < 0.0001 for systolic and diastolic BP, respectively) [30].

A recent randomized trial assessed the hemodynamic effects of intravenous paracetamol in healthy subjects, providing a different scenario. Infusion of paracetamol was found to induce a transient but significant fall in mean BP (− 1.85 mmHg) associated with reduced systemic vascular resistance and increased cardiac index, thus suggesting vasodilation [31•]. Similarly, hypotension following paracetamol infusion has been reported in critically ill patients, with some authors hypothesizing a reduction in cardiac index [32, 33•].

In conclusion, the effects of paracetamol on BP are still controversial. Randomized data from larger samples are needed and evidence specifically referring to hypertensive patients should be implemented. In addition, the effects of paracetamol on out-of-office BP should be investigated, as well as its potential interactions with antihypertensive medications.

### Non-Steroidal Anti-Inflammatory Drugs (NSAIDs) and COX-2 Inhibitors

It is widely recognized that non-steroidal anti-inflammatory drugs (NSAIDs) may increase BP values, particularly in hypertensive patients [24, 34]. A meta-analysis by Pope et al. including 1324 patients (mean age 46, 92% hypertensive) reported a 3.3 mmHg mean arterial pressure increase associated with NSAIDs use [35]. After adjusting for sodium intake, naproxen and indomethacin were associated with the largest BP increase (+ 3.7 and + 3.6 mmHg, respectively), while piroxicam, aspirin, and ibuprofen had negligible pressor effects [35]. A meta-analysis by Johnson et al. reported similar results, describing a 5 mmHg mean arterial pressure increase in hypertensive patients receiving NSAIDs [36]. Piroxicam was associated with the highest BP increase when compared to placebo (+ 6.2 mmHg), while aspirin had minimal BP effects [36]. Consistently, in a previous study involving 18,790 hypertensive individuals, aspirin 75 mg daily was proved to have no interference with antihypertensive therapy [37].

Of notice, although NSAIDs are reported to have reduced BP effects in normotensive individuals [38, 39], some studies reveal an increased risk of incident hypertension associated with NSAIDs use [20, 40].

BP changes may also occur in patients receiving selective COX-2 inhibitors, with particular reference to etoricoxib and rofecoxib (the latter now withdrawn from the market) [39, 41, 42]. By contrast, celecoxib seems to lesser impact office and ambulatory BP values compared to other selective and non-selective NSAIDs [34, 42, 43•, 44]. Similar to paracetamol, the BP effects of NSAIDs are supposed to be related to the inhibition of the COX pathway. Two isoforms of COX enzymes exist, commonly referred to as COX-1 and COX-2. The first is constitutively expressed in most tissues, while the second is mainly upregulated with inflammation and cell injury. Selective NSAIDs inhibit the COX-2 isoform, while other NSAIDs may preferentially inhibit COX-1 or have a balanced effect. Experimental studies on animals confirm that inhibition of both COX enzymes may lead to BP increase [45], thus providing a physiopathological explanation for the BP effects of NSAIDs. In addition, it has been suggested that other mechanisms might contribute to NSAIDs-mediated BP elevation, including an increase in endothelin-1 production and aldosterone levels [46, 47]. The pressor effects of NSAIDs may be more common in older people, due to an age-related susceptibility to salt retention which is further exacerbated by the inhibition of PGs synthesis [39, 48]. Unlike other NSAIDs, celecoxib inhibits calcium responses in vascular smooth muscle and reduces vascular tone, independent of COX-2 inhibition. This pharmacodynamic characteristic may provide an explanation for the limited impact of celecoxib on BP, as this mechanism probably counterbalances the increase in vasoconstriction which is induced by COX-2 inhibition [49].

It should be considered that PGs are involved in the pharmacodynamics of some antihypertensive medications, e.g., their natriuretic effect is complementary to that of diuretics, while ACE-inhibitors effects are at least partly mediated by bradykinin, a vasodilating molecule acting through the induction of PGs release [50]. Consequently, ACE-inhibitors, angiotensin receptor blockers, and diuretics seem to be affected most by NSAIDs co-administration [51•, 52]. The antihypertensive response to calcium antagonists does not seem to be attenuated instead [39, 53, 54], even if an interaction with NSAIDs is reported by some authors [51•]. Interaction of NSAIDs with beta-blockers still remains unclear [51•].

In conclusion, available data indicate that patients should be monitored for BP changes when initiating NSAID treatment, with particular reference to older and hypertensive individuals. Studies assessing the impact of NSAIDs on BP have mainly focused on young and relatively healthy individuals with controlled hypertension, while data on older patients with multimorbidity and/or uncontrolled hypertension are lacking [9••].

### Opioids

As concerns the hemodynamic effects of opioid drugs, available literature mainly refers to acute intravenous administration during anesthesia or postoperative analgesia. In this context, opioids may cause relevant cardiovascular effects, including hypotension and bradycardia, particularly if benzodiazepines are co-administered [55•]. Data on chronic opioid treatment are limited, but hypotension, orthostatic hypotension, and syncope are commonly reported among potential adverse effects of most opioid analgesics, such as morphine, buprenorphine, fentanyl, oxycodone, and tapentadol [55•]. Yet, the mechanism underlying opioid-mediated hypotension still remains a matter of debate.

As many opioids are potent histamine releasers [56], their hypotensive effects may result from histamine-mediated vasodilation. However, if histamine release has been clearly demonstrated for morphine, codeine, and pethidine [57–59], it is reported to be minimal or absent for oxycodone and fentanyl [57, 60, 61]. In addition to histamine effects, opioid-induced hypotension may also derive from an attenuation of sympathetic alpha-adrenergic outflow, leading to peripheral vasodilation [62]. Finally, the release of nitric oxide and the activation of vagal reflex have been hypothesized as alternative mechanisms [63, 64]. Hypotensive effects might be more relevant in the presence of hypertension, due to a hypersensitivity to opioid receptor agonists or an overexpression of opioid receptors in hypertensive people [65].

### Adjuvant Analgesics

*Antidepressants* are increasingly used as an adjuvant therapy for chronic pain, particularly in patients with neuropathic pain, migraine, and fibromyalgia. It is known that antidepressants may influence BP values, but different effects are reported according to the drug class considered.

Tricyclic antidepressants (TCA) and serotonin-norepinephrine reuptake inhibitors (SNRI) are traditionally considered to have hypertensive effects and were shown to be associated with an increased risk of incident hypertension [66•, 67••]. The BP effects of SNRI seem to be dose-dependent and become more relevant at supratherapeutic doses [68, 69]. As proof of that, some studies observed no significant BP changes in patients receiving venlafaxine or duloxetine at therapeutic doses [70, 71].

The potentiation of adrenergic transmission may account for the association of TCA and SNRI with increased BP, especially when considering that the majority of norepinephrine which is released in the heart is recaptured into sympathetic nerves. The inhibition of norepinephrine reuptake by TCA and SNRI could thus result in increased cardiac sensitivity to sympathetic stimulation [72]. In addition to the above, the anticholinergic action of TCA may further contribute to BP elevation [73•].

The BP effects of selective serotonin reuptake inhibitors (SSRIs) are currently unclear. Experimental studies indicate that SSRIs may potentially lower BP and a vasodilating effect has been reported for fluoxetine, citalopram, and sertraline, which is likely mediated by the inhibition of calcium-elicited vasoconstriction [74–76]. Moreover, fluoxetine and paroxetine act as cytochrome CYP2D6 inhibitors, thus potentially reducing the metabolic rate of hypotensive drugs like nifedipine and beta-blockers [77]. Finally, some authors suggest that SSRIs may attenuate sympathetic activity [78]. However, SSRIs were not found to affect BP values in a recent review and meta-analysis [67••].

We may conclude that TCA and SNRI could have pro-hypertensive effects, whereas the impact of SSRI on BP has yet to be clarified. Available studies mainly involved patients with depression, which itself has been reported to be associated with an increased risk of hypertension [79], thus making it more difficult to interpret the association between antidepressants and BP. Future studies should preferably explore the BP effects of antidepressants when prescribed in normotensive and hypertensive adults with chronic pain.

Along with antidepressants, *anticonvulsants* represent an important adjuvant for pain management, particularly in patients with neuropathic pain and fibromyalgia. Gabapentinoids such as gabapentin are the molecules with the strongest evidence for chronic pain treatment, while carbamazepine is mainly effective in trigeminal neuralgia. It has been recently demonstrated that gabapentin can induce vasodepression and bradycardia acting through the nitric oxide pathway [80]. Indeed, gabapentin is known to attenuate the hypertensive response to laryngoscopy and tracheal intubation [81].

Evidence concerning the effects of carbamazepine on BP is scarce. Some case reports describe severe uncontrolled hypertension induced by carbamazepine, which may derive from antagonism of central noradrenergic transmission [82–84]. In addition, it should be recalled that carbamazepine is a potent CYP3A4 inducer and may decrease plasma concentration of antihypertensive medications via cytochrome induction. The latter mechanism may be particularly relevant for calcium antagonists [85].

As chronic pain seems to negatively influence BP regulation, we could expect that pain relief deriving from analgesic treatment might instead favor BP control in hypertensive patients. Consequently, patients receiving effective analgesic therapy should present with better BP control as compared to patients with untreated or poorly controlled chronic pain. Yet, the above-described direct effects of analgesics on BP may act as a confounder, thus making it difficult to assess whether pain therapy improves BP control in hypertensive individuals. However, the BP effects of pain relief have not been investigated to date and specific evidence is lacking.

## Conclusions and Perspectives

Pain and blood pressure appear to be strictly related. According to available evidence, both pain and analgesic therapies may induce a clinically significant destabilization of blood pressure values. The subsequent implications on hypertension incidence and blood pressure control remain unclear and should be explored in future studies.

## Data Availability

Not applicable.
